# Severe Mucus Plugging Causing Acute Hypoxic Respiratory Failure and Delayed Hemoptysis in a Renal Transplant Recipient Without Chronic Pulmonary Disease

**DOI:** 10.7759/cureus.108907

**Published:** 2026-05-15

**Authors:** Sitha Konopack

**Affiliations:** 1 Department of Emergency Medicine, University of Florida Health, Gainesville, USA

**Keywords:** airway obstruction, bronchoscopy, hemoptysis, mucus plugging, post-intubation complications

## Abstract

Mucus plugging is typically associated with chronic pulmonary diseases such as asthma, chronic obstructive pulmonary disease, and allergic bronchopulmonary aspergillosis. Clinically significant mucus plugging leading to acute respiratory failure in patients without underlying lung disease is rare. We present the case of a 36-year-old female patient with a history of renal transplantation who developed acute hypoxic respiratory failure requiring intubation due to extensive mucus plugging, followed by recurrent hemoptysis after extubation. Repeat bronchoscopy revealed significant tracheal granulation tissue and infection with methicillin-sensitive *Staphylococcus aureus*. This case highlights the potential for severe airway obstruction and delayed postintubation complications in patients without known chronic pulmonary conditions, particularly in the setting of recent intubation and immunosuppression.

## Introduction

Mucus plugging results from the accumulation of inspissated secretions within the airways, leading to obstruction and impaired ventilation. It is most commonly associated with chronic pulmonary conditions such as asthma, chronic obstructive pulmonary disease (COPD), allergic bronchopulmonary aspergillosis, and plastic bronchitis [1].

Clinically significant mucus plugging in patients without underlying chronic lung disease is rarely reported [1,2]. When present, it may result in acute hypoxic respiratory failure requiring urgent airway intervention [3]. Additionally, airway injury following endotracheal intubation may predispose patients to impaired mucociliary clearance, granulation tissue formation, and subsequent airway compromise [4-6].

We describe a case of severe mucus plugging causing acute respiratory failure in a renal transplant recipient without underlying chronic pulmonary disease, followed by delayed hemoptysis associated with tracheal granulation tissue and superimposed infection after extubation.

## Case presentation

A 36-year-old female patient with a past medical history of end-stage renal disease secondary to uncontrolled hypertension, status post renal transplant in 2019, presented initially with shortness of breath, chest pain, and hypoxia. On arrival, she was tachypneic (31 breaths/minute), was diaphoretic, and in significant respiratory distress, necessitating endotracheal intubation. Her blood pressure was 238/119 mmHg.

**Figure 1:**
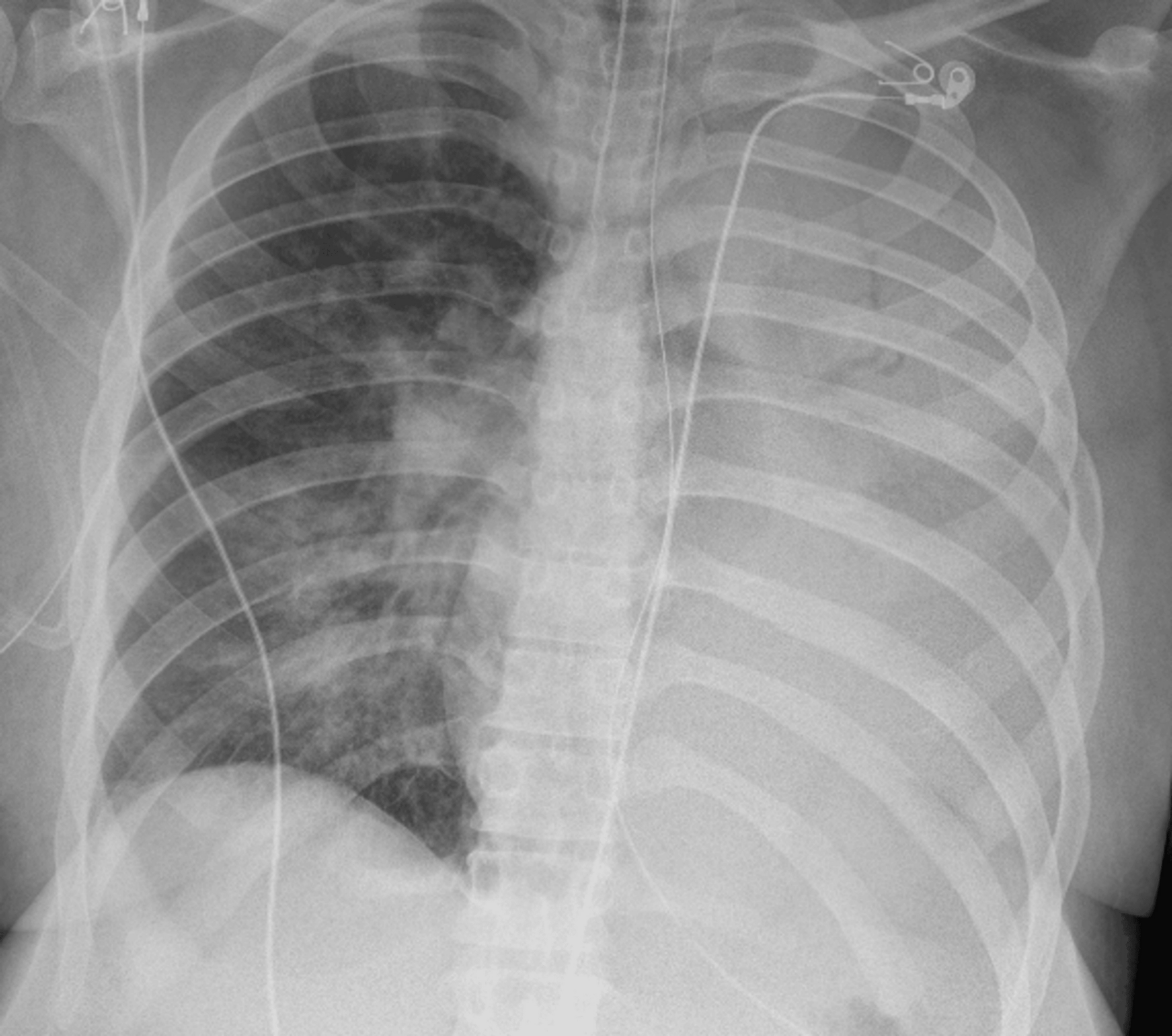
Near-complete white-out of one hemithorax Chest radiograph demonstrating complete opacification of the left hemithorax with mediastinal shift toward the left, consistent with collapse of the left lung secondary to airway obstruction

**Figure 2:**
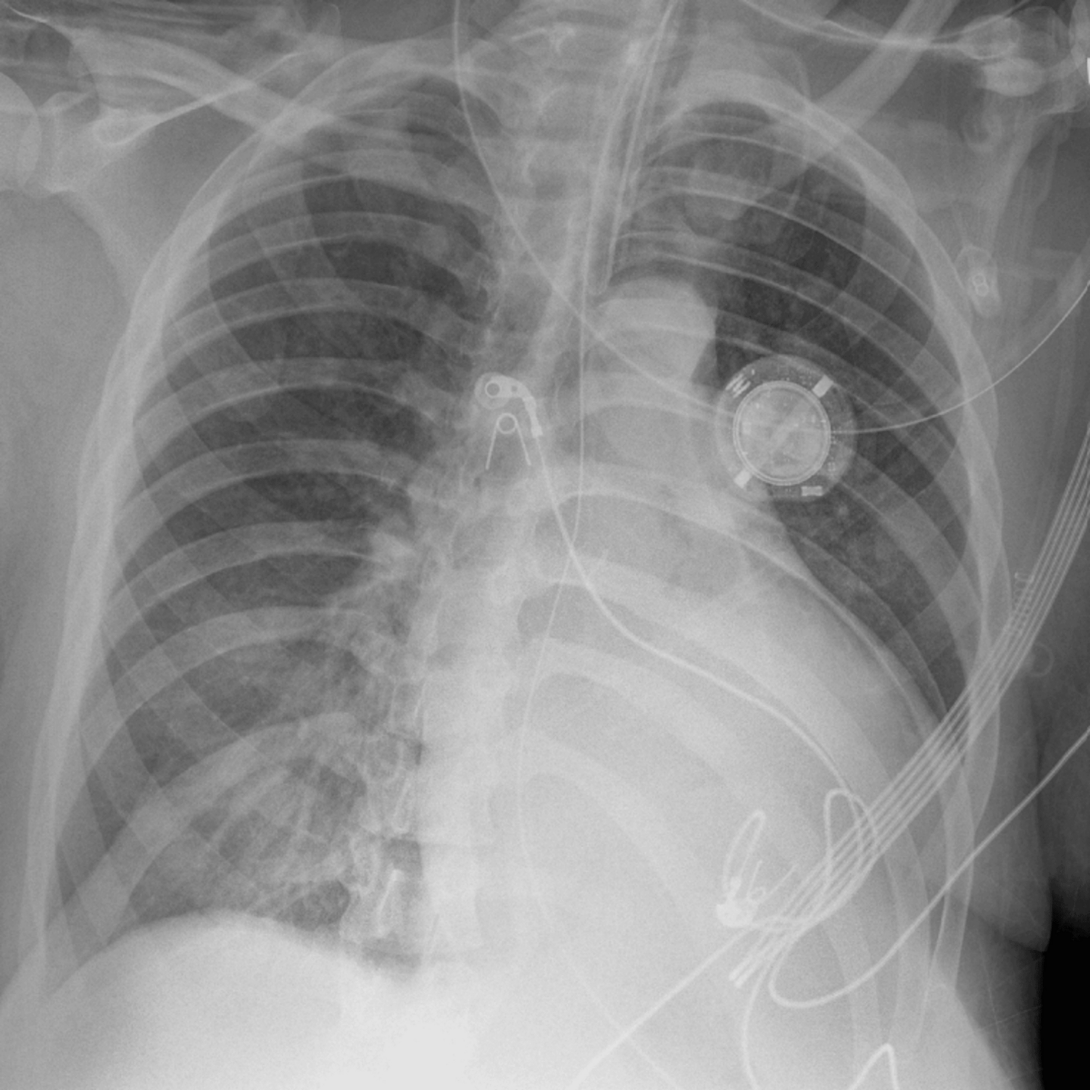
Reexpansion after bronchoscopic airway clearance Chest radiograph showing reexpansion of the left lung and correction of mediastinal shift following removal of extensive mucus plugging

Chest radiography obtained after intubation demonstrated complete opacification of the left hemithorax with associated mediastinal shift toward the left, concerning for complete lung collapse secondary to central airway obstruction (Figure 1). Flexible bronchoscopy revealed extensive mucus plugging obstructing the left-sided airways. Therapeutic airway clearance resulted in immediate improvement in ventilation and radiographic reexpansion of the left lung on repeat imaging (Figure 2). The patient was subsequently extubated and discharged in stable condition with a diagnosis of acute hypoxemic respiratory failure of unclear etiology.

**Figure 3:**
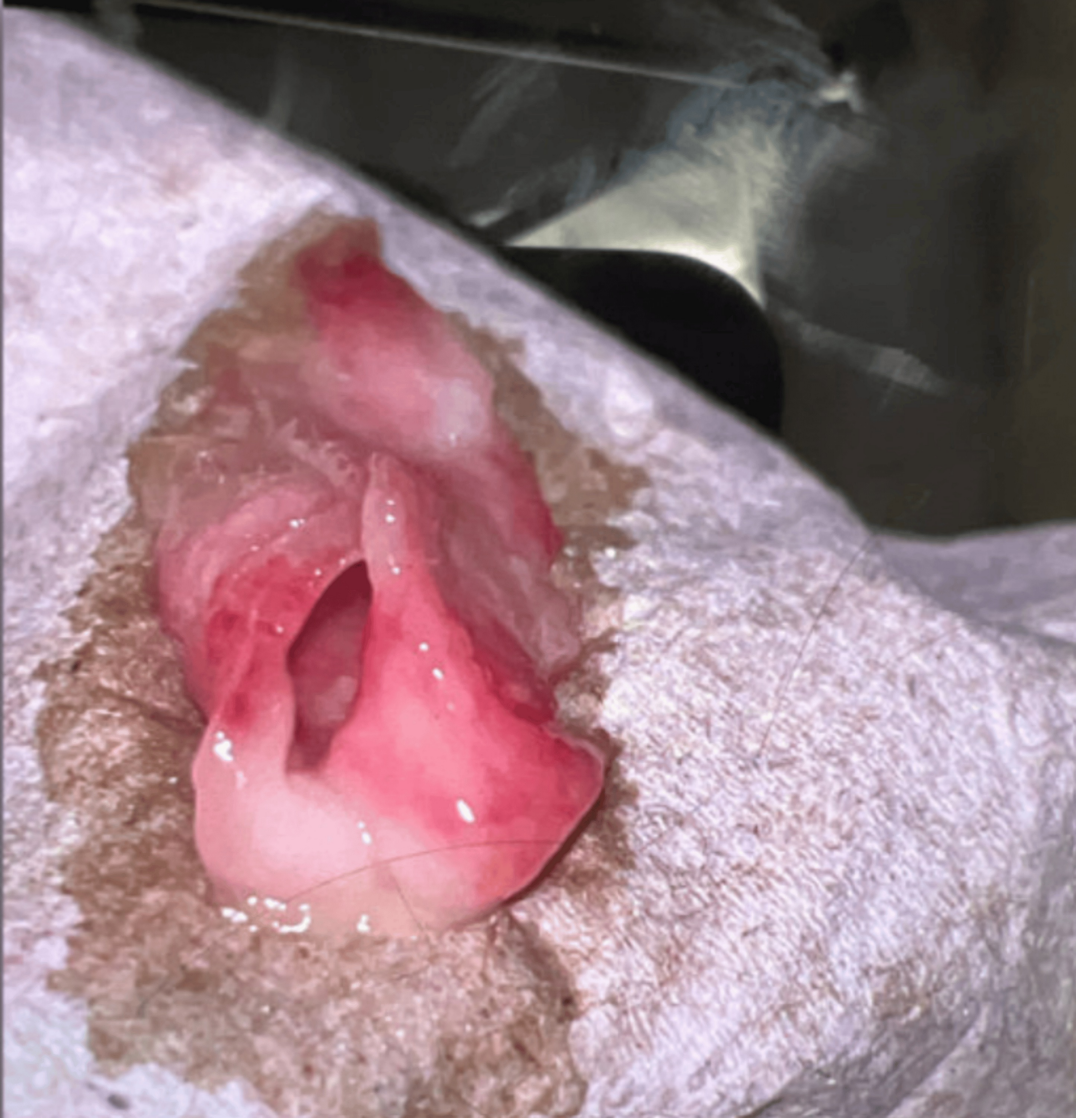
Expectorated material following hemoptysis Large, thick inspissated secretions and clot burden expectorated by the patient following recurrent airway obstruction

**Figure 4:**
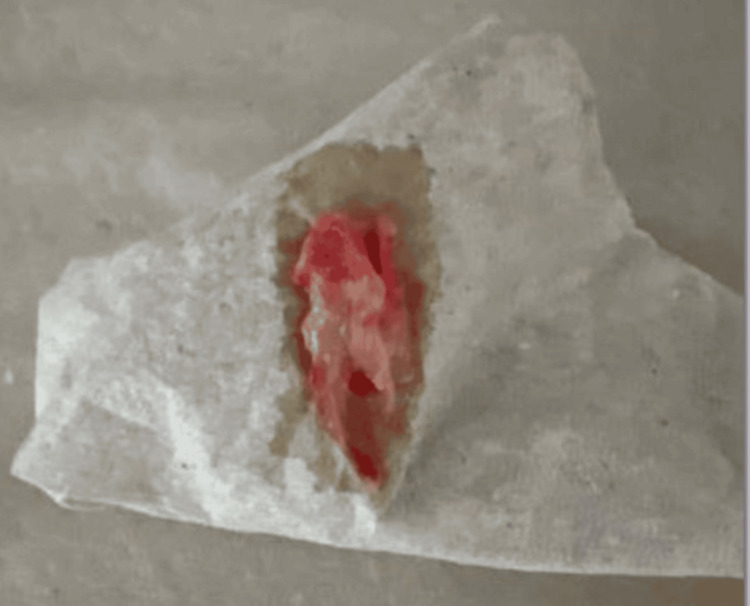
Thick bronchial secretions consistent with mucus plugging Partially expectorated bronchial casts and inspissated secretions contributing to airway obstruction

Five days after extubation, the patient re-presented to a freestanding emergency department with a 24-hour history of large-volume hemoptysis, including expectoration of multiple quarter-sized blood clots and tissue-like material (Figures 3, 4). Vital signs were stable, and she was maintaining oxygen saturation on room air.

**Figure 5:**
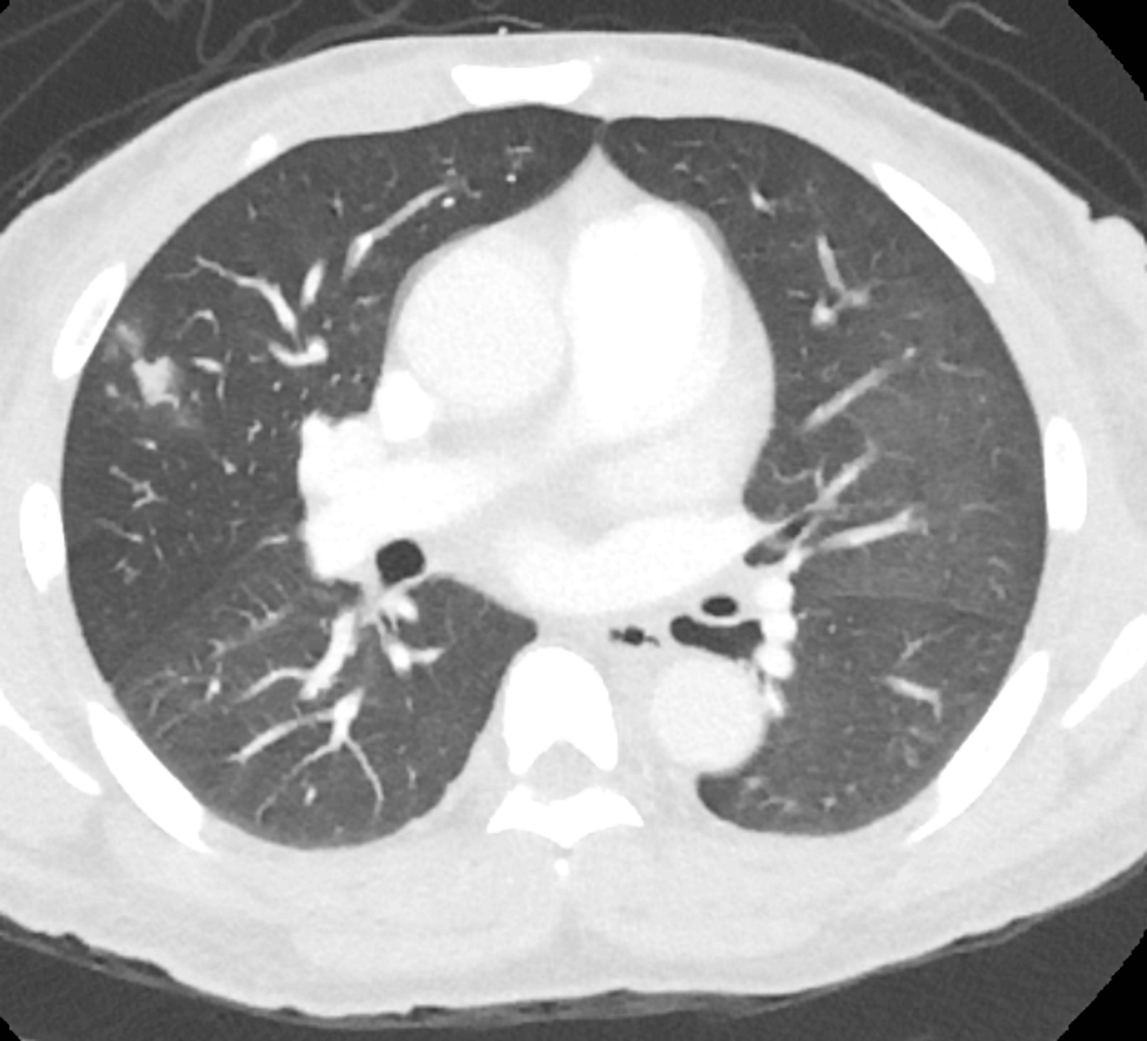
Computed tomography showing multifocal ground-glass and patchy consolidative opacities Computed tomography pulmonary angiography demonstrating multifocal ground-glass and patchy consolidative opacities without evidence of pulmonary embolism

Computed tomography pulmonary angiography showed interval development of new patchy airspace opacities without evidence of pulmonary embolism (Figure 5). Due to continued hemoptysis, she was admitted to the intensive care unit for further evaluation.

Repeat bronchoscopy demonstrated extensive granulation tissue involving multiple proximal tracheal rings. Bronchoalveolar lavage was performed in the right middle lobe without evidence of progressively bloodier return, making diffuse alveolar hemorrhage less likely. A pneumonia panel was positive for methicillin-sensitive *Staphylococcus aureus* (MSSA).

Sputum culture demonstrated no growth, with no white blood cells or organisms identified. Histopathologic evaluation of the expectorated sputum/membranous tissue specimen (Figure 3) revealed a 1.7 cm x 0.5 cm x 0.3 cm tan-pink, mildly indurated membranous tissue fragment on gross examination. Microscopic examination demonstrated inflammatory debris composed of fibrin, acute inflammatory cells, and associated bacterial colonies.

The patient improved with supportive care and antimicrobial therapy and was discharged in stable condition on levofloxacin and nifedipine, with outpatient follow-up arranged with interventional pulmonology.

## Discussion

Mucus plugging is classically associated with chronic inflammatory airway diseases such as asthma, COPD, allergic bronchopulmonary aspergillosis, and cystic fibrosis [1-3]. However, severe mucus plugging resulting in acute respiratory failure in patients without established pulmonary disease remains uncommon.

In this case, several factors likely contributed to impaired secretion clearance and airway obstruction, including recent endotracheal intubation, immunosuppression following renal transplantation, and superimposed infection. Endotracheal intubation is known to cause mucosal injury, local inflammation, disruption of mucociliary function, and abnormal wound healing, all of which may predispose to secretion retention and granulation tissue formation [4-6].

The extensive tracheal granulation tissue identified on repeat bronchoscopy likely contributed to both recurrent mucus retention and hemoptysis. Mechanical trauma from intubation may lead to tracheal ulceration and excessive granulation tissue proliferation, particularly in susceptible patients [4,5]. Although postintubation airway complications more commonly present as tracheal stenosis, this case demonstrates that clinically significant mucus retention and delayed hemoptysis may also occur [6].

Superimposed infection may have further exacerbated airway inflammation and secretion burden. Detection of MSSA on the molecular pneumonia panel suggests an infectious contribution to the patient’s ongoing respiratory symptoms and airway irritation [7].

The differential diagnosis for massive hemoptysis in this patient included diffuse alveolar hemorrhage, pneumonia, pulmonary embolism, bronchitis, and airway trauma. Pulmonary embolism was excluded on computed tomography pulmonary angiography, while bronchoalveolar lavage findings argued against diffuse alveolar hemorrhage. The constellation of bronchoscopic findings, expectorated inflammatory debris, and tracheal granulation tissue favored airway injury with retained secretions as the primary mechanism of bleeding.

This case underscores the importance of considering mucus plugging and postintubation airway pathology even in patients without underlying chronic lung disease, particularly in immunocompromised individuals presenting with recurrent respiratory symptoms after extubation. Early bronchoscopy may be both diagnostic and therapeutic in such cases.

## Conclusions

Clinically significant mucus plugging can occur in patients without underlying chronic pulmonary disease, particularly in the setting of recent intubation and immunosuppression. This case illustrates the potential for acute respiratory failure followed by delayed complications such as hemoptysis and tracheal granulation tissue formation after extubation. Early recognition, airway clearance, and consideration of repeat bronchoscopy are essential in managing similar presentations.
